# Determining staffing needs for improving primary health care service delivery in Kaduna State, Nigeria

**DOI:** 10.12688/f1000research.110039.1

**Published:** 2022-04-19

**Authors:** Agbonkhese I. Oaiya, Oluwabambi Tinuoye, Layi Olatawura, Hadiza Balarabe, Hamza Abubakar

**Affiliations:** 1 PATH, Abuja, Nigeria; 2 Abts Associates, FCT Abuja, Nigeria; 3 Health Strategy and Delivery Foundation, FCT Abuja, Nigeria; 4 Kaduna State Government House, Kaduna, Nigeria; 5 Kaduna State Primary Health Care Board, Kaduna, Nigeria

**Keywords:** Universal Health Coverage; Health Workforce; Human Resources for Health; Workload Indicators for Staffing Need; Nigeria; Service Delivery; Nurse; Midwife; Community Health Officer; Community Health Extension Worker; Junior Community Health Extension Worker.

## Abstract

**Background:** The equitable distribution of a skilled health workforce is critical to health service delivery, and Kaduna state has taken significant steps to revamp the primary health care system to ensure access to health care for its populace. However, some of these investments are yet to yield the desired outcomes due to workforce shortages and inequitable distribution of those available.

**Methods:** A Workload Indicator for Staffing Need study was conducted at the primary health care level in Kaduna state. The study focused on estimating staffing requirements; Nurse/Midwife and Community Health Worker practitioners; Community Health Officer, Community Health Extension Worker and Junior Community Health Extension Worker, in all government prioritized primary health care facilities. Ten focal primary health care facilities in Kaduna North Local Government Area were included in the study.

**Results:** Findings revealed a shortage of Nurses/Midwives and Community Health Workers across the study facilities. For the Nurse/Midwife staffing category, 9/10 PHCs have a Workload Indicator for Staffing Need ratio < 1; indicating that the number of staff in the Nurse/Midwife category is insufficient to cope with the workload. In two of the ten primary health care facilities, there is an excess in the number of CHWs available; a Workload Indicator for Staffing Need ratio > 1 was calculated.

**Conclusions:** The Workload Indicator for Staffing Need study highlights the staffing needs in government prioritized primary health care facilities in Kaduna state. This evidence establishes the basis for the application of an evidence-based approach to determining staffing needs across the primary health care sector in the State, to guide workforce planning strategies and future investments in the health sector. The World Health Organisation Workload Indicator for Staffing Need tool is useful in estimating staffing needs required to cope with workload pressures, particularly in a resources-constrained environment like Kaduna State.

## Introduction

The equitable distribution of resources, specifically Human Resources for Health (HRH), to meet the needs of the populace is critical for achieving Universal Health Coverage (UHC). However, this critical health system component is plagued with challenges of availability, distribution, skills, and retention. These challenges are prominent in developing countries like Nigeria, where shortages and inequitable distribution of health workforce, poor HRH planning, inadequate recruitment exacerbated by a ban on workforce recruitment, and weak retention strategies, are often encountered. ^1^^–^^4^ These frequently result in disparities in health worker densities by geographic location; urban and rural areas, limit access to health care services and inadvertently undermine the quality of care provided all of which are associated with poor health outcomes.

In the last decade, Nigeria has developed some health system reforms aimed at improving national health indices. One of such reforms is the Primary Health Care (PHC) Minimum Service Package (MSP). ^5^^,^^6^ The MSP documents services to be provided by primary health facilities, articulates recommendations on staffing norms and composition, as well as states what services each health worker cadre should provide to meet the health needs of the populace. ^5^^,^^6^ The MSP strategy was particularly important because it was birthed in wake of the UHC push in Nigeria and it targets primary healthcare which is the entry point into the health system, where promotive, preventive, and curative services for uncomplicated minor ailments are provided in Nigeria. ^5^^–^^7^ The MSP stipulates that at the primary care level, the health workforce should include medical officers, nurses, midwives, community health practitioners, laboratory technicians, pharmacy technicians, health records assistants and environmental health officers. ^5^^–^^7^ However, these staffing standards have remained unmet. This may be due to the health workforce crisis that Nigeria is facing as well as the low government spending on health at national and sub-national levels. ^8^ In response to sub-optimal staffing patterns, the Government of Nigeria developed a Task Shifting and Task Sharing (TSTS) policy that allows lower skilled clinical staff to perform high skilled clinical tasks following training. ^9^ However, there are challenges with the implementation and monitoring of this policy.

Kaduna is a state in northern Nigeria, with a projected population of 8.98 million people. ^10^ Kaduna is one of the 36 states in Nigeria that have made huge investments in healthcare. ^11^ To attain UHC through community participation, as contained in the national health act, the State adopted and implemented the Ward Health System (WHS) for healthcare. ^5^^–^^7^^,^^12^ The WHS utilises the electoral wards as the basic operational unit for PHC service delivery. This formed the basis for governments' investment in one PHC facility per ward. There are 255 wards in Kaduna state, and in each ward, the State government prioritized one PHC facility. Since 2015, 255 PHC facilities have benefitted from the government’s investments in establishing and sustaining a multi-level administrative governance structure, improved infrastructure, provision of basic equipment and essential medicines amongst others. However, there have been challenges in meeting the staffing needs for these facilities as stipulated by the MSP because of the State’s limited fiscal space for health spending. As such, the availability of a sufficient, skilled, and equitably distributed workforce to serve the population has remained a challenge.

In consideration of Kaduna State’s HRH gaps, and an alternative to the staffing norms stipulated in the MSP, there is a need to employ an evidence-based staffing approach that can support the determination of adequate staffing norms in line with existing health system complexities. One of such methods is the Workload Indicators for Staffing Need (WISN) method developed by the World Health Organization (WHO). This study aimed to estimate the staffing requirements for delivery of care at focal primary health facilities by health worker cadre in Kaduna North Local Government Area in Kaduna State, Nigeria employing the WISN methodology.

## Methods

### Ethics approval and consent to participate

Written informed consent was obtained before data collection during the field visit and focus group discussion, and through the Health Research Ethics Committee (HREC) of the Kaduna State Ministry of Health has an approved registration number NHREC/17/03/2018.

This study employed the WISN methodology to determine staffing needs. WISN is designed by the WHO and supports the evidential determination of the number of health workers by cadre required to cope with the workload in a particular health facility. The WISN methodology considers several relevant components by health worker cadre that includes: (i) services delivered (ii) the time it takes to deliver both clinical and non-clinical services (iii) the total annual work time available to each Health Care Workers (HCW) cadre as well as (iv) retrospective annual service delivery statistics in the health facility. ^13^ Computation of these statistics produces a determined number of HCWs by cadre required in the health facility.

### Scope of the study

The WISN study was completed in Kaduna North Local Government Area and included ten (10) primary health facilities. The study population were clinical health workers available and tasked with providing healthcare services to patients at these primary health facilities. These prioritized cadres are Nurse/Midwives and Community Health Workers (CHW), comprising Community Health Officers (CHOs), Community Health Extension Workers (CHEWs) and Junior Community Health Extension Workers (JCHEWS). Reproductive Maternal and Newborn Child Health (RMNCH) services are predominantly provided at the primary care level which makes up most of the health facility visits in the LGA and these services were prioritized for the study.

### Establishing state governance structures

Three Technical Working Groups (TWGs); Steering Committee, Technical Task Force and Expert Group were inaugurated to conduct the study. These study groups were a subset of the States’ larger HRH TWG, whose objectives include providing advisory and technical support to the state government in workforce policy formulation and technical directions to enable the development of the HRH workforce in the state. The membership composition of the three groups was drawn from relevant Ministries, Departments and Agencies (MDA), health training institutions, Civil Society Organisations (CSO), health facility heads and development partners. These groups were engaged to build local capacity, decide on priority areas of primary healthcare for the state as well as create the utility of study results for workforce planning.

### Health facility inclusion criteria

Kaduna North Local Government Areas (LGA) was selected for the study for convenience. Consequently, all government prioritized PHC facilities that have been in operation for at least one year before the time of the study were included. Kaduna North LGA is an urban area and one of the most populous areas in the State. The choice of including only government prioritized PHC facilities is hinged on the significant investments made by the State government and donors in these facilities and a resultant increase in service utilization rates.

### Data collection

After a review of relevant documents that include the Nigeria Task Shifting and Task Sharing (TSTS) policy, the MSP, Ward Minimum Healthcare Package (WMHCP) and the public service handbook, data collection tools were developed. Service delivery statistics and HRH composition data were extracted manually from secondary sources that include the Nigeria District Health Information System (DHIS2) and KSPHCB Human Resources for Health Information System (HRH-IS) respectively, and tri-angulated during primary data collection. Staff available work time data and activity standards data for both clinical and non-clinical health services were obtained following a focus group discussion with an Expert Group (EG).

Clinical health care services within the Reproductive Maternal and New-born Child Health (RMNCH) continuum of care formed the core health services assessed for this study because they are the commonly provided and accessed services within PHC. These services include family planning, antenatal care, post-natal care, immunization, diarrhoea, pneumonia and malaria in children and adults because of their endemicity. Annual RMNCH health service statistics, January to December 2019, were obtained from the national DHIS2. The DHIS2 is the electronic instance of the National Health Management Information System (NHMIS); a paper-based mechanism that aggregates all health care services delivered in a health facility. The annual statistics data collected from the DHIS2 were compared and triangulated with data obtained from the health facility registers during field visits that ran for three weeks between June and July 2021.

Facility workforce data focusing on clinical professional cadre – Nurse/Midwife, CHWs, CHO, CHEW and JCHEW – were obtained from two sources; HRH-IS domiciled in the KSPHCB as well as health facility staff register to facilitate triangulation.

To obtain information on staff Available Work Time (AWT), the total amount of time available to an HCW by cadre to perform daily tasks in a year considering authorized and unauthorized absences, a multi-step approach was taken. Firstly, a desk review of relevant public service statutory policy, rules, and guidelines; public service handbook, as well as other grey literature was conducted to obtain HCW’s working hours per day, working days per week, and authorized and unauthorized absences allowed within the service. Only resources relevant to public sector workforce administration were included in the desk review. The Staff AWT was subsequently reviewed and approved by the study’s governance structure.

An Expert Group comprising 17 clinical experts was convened to obtain time spent by HCWs in the study’s cadre of interest on both clinical and non-clinical activities. These experts were purposefully selected and are currently employed in the public service possessing at least 15 years of experience providing health care services at the primary care level. All experts included in the group responded on the time it takes the prioritised health worker cadre to perform these activities to acceptable standards and the mean value of their responses was utilized.

### Data analysis and interpretation

The data collected were analysed using MS Excel, consistent with the WISN methodology. Activity standards clinical and non-clinical workload components, annual service delivery statistics and AWT for the prioritized cadre for each facility were included. To complete computation, the data collected was defined and analysed as follows:

• Available Working Time: The time a health worker is available in one year to do his or her work, considering authorized and unauthorized absences. AWT in Days is the difference between Possible Working days in a year (PWD) and Non-working days in a year (authorized and unauthorized absences). ^13^^–^^15^

$$\mathrm{AWT}=A–\left(B+C+D+E\right)$$ (1.0)

Where in the formula:

AWT is the total staff available working time

A is the number of possible working days in a year

B is the number of days off for public holidays in a year

C is the number of days off for official leave in a year

D is the number of days off due to sick leave in a year

E is the number of days off due to casual leave, study or training leave and maternity leave in a year.

• Activity Standard: The time it takes an HCW of a particular cadre to deliver both clinical and non-clinical services; core, individual and support activities. ^13^
• Standard Workload: The amount of work one HCW can perform in a year within a health service category. ^13^ It is calculated in unit time or rate of work, by dividing AWT by the time taken to conduct the work or multiplying the AWT with the rate of working respectively.
• Core health activities: These are clinical health services. This refers to activities directly related to service delivery performed by all staff of a cadre.
• Support activities: There are non-clinical activities performed by a health worker but not directly related to a patient and usually involve all staff of the same cadre.
• Additional activities: These activities are also not clinically related and are performed by health workers but not directly related to a patient. They usually do not involve all staff of the same cadre.
• Staff requirement for core health activities: This was calculated by taking the aggregate ratio of all annual core health services and standard workload for the identified clinical health services:
$$\sum_{i}^{n}\left(\frac{\mathrm{AWi}}{\mathrm{SWi}}\right)$$ (2.0)
  Core health activities i = 1,2,3 … n
  AWi = Annual statistics for each core clinical health service
  SWi = Standard Workload for each core clinical health service
• Staff requirement for support activities: A Category Allowance Standard (CAS) which is the percentage of the working time required to cope with all support activities was estimated and Category Allowance Factor (CAF) was calculated using:
$$\mathrm{CAF}=100/\left(100-\text{Total}\;\mathrm{CAS}\right)$$ (3.0)
  Staff requirement for individual activities: Individual Allowance Standard (IAS) which is the total number of hours per year needed to perform all additional activities undertaken by some HCWs were also calculated. An Individual Allowance Factor (IAF) identifying the staffing requirement to undertake these workloads was estimated using:
IAF=TotalIAS÷AWT (4.0)
• Total staffing requirement was calculated using:
$$\text{Total WISN Staff Requirement}=\mathrm{CAF}*\sum_{i}^{n}\left(\frac{\mathrm{AWi}}{\mathrm{SWi}}\right)+\mathrm{IAF}$$ (5.0)

WISN staffing results with fractions were handled as recommended by the WISN guide. ^13^ WISN differences and ratios were also generated. The WISN difference, which is calculated from the variance between the current staffing norm available by cadre and the computed staffing requirements identifies staffing gaps or excesses by cadre. The ratio represents the work pressure experienced by the HCW. A WISN ratio of > 1 indicates the availability of more HCWs than required to meet the facility workload.

## Results

### Clinical and non-clinical workload components and standards

Two categories of health care services were included in the study. The clinical health service forms the core health activities, while the non-clinical services comprise both support and additional services. The clinical core health services refer to activities directly related to service delivery performed by all prioritized cadres. ^13^^–^^15^ Support activities are part of the non-clinical category, and activities performed by the prioritized cadre are not directly related to patient care and usually involve all staff of the same cadre. ^13^^–^^15^ Additional activities are activities performed by these prioritized cadres that are not directly related to patient care and are undertaken by just a staff. ^13^^–^^15^

The workload and activity standards developed and validated by the expert group are presented in Table 1.

**Table 1.** Clinical and non-clinical workload components and service standards for primary level of care.

Service activity standards	Nurse/midwife	CHW	Unit
**A) Clinical health service – core health service activity standards**			
Antenatal care	10	10	Minutes/Client
Delivery	60	60	Minutes/Client
Postnatal care	14	19	Minutes/Client
Family planning (counselling)	18	17	Minutes/Client
Family planning (insertion of IUCD)	20	17	Minutes/Client
Family planning (insertion of implant)	17	13	Minutes/Client
Family planning (injection)	7	8	Minutes/Client
Family planning (oral pills)	7	9	Minutes/Client
Immunization	9	11	Minutes/Client
Diarrhoea in U5 years old	14	13	Minutes/Client
Pneumonia in U5 years old	15	14	Minutes/Client
Confirmed uncomplicated malaria	18	21	Minutes/Client
**B) Non-clinical - support services activity standards**			
Community mobilization and education	0	2	Hours/Week
Ward development committee meetings	51	50	Minutes/Month
Outreaches/community-based services	0	2	Hours/Week
Hand over/report writing	36	25	Minutes/Week
Staff meetings	38	33	Minutes/Week
Documentation on patients	3	3	Minutes
Group health education	40	31	Minutes/Week
**C) Non-clinical - individual service activity standards**			
Supervision of students	60	60	Minutes/Day
General administration	50	66	Minutes/Day
Monthly report writing	50	69	Minutes/Month
Review meetings	2	3	Hours/Month
Mentoring of subordinates	44	36	Minutes/Day
Facility management meeting	51	39	Minutes/Month
Sterilization of equipment	5	4	Minutes/Day

CHW – Community Health Worker Practitioners comprising of CHO – Community Health Officers; CHEW – Community Health Extension Workers; JCHEW – Junior Community Health Extension Workers.

A total of 26 health and non-health related services were identified in the state Primary Health Care level; of which 12 are clinical/core health services conducted by both Nurse/Midwife and CHW Practitioners. 14 non-clinical services; 7 support services and 7 individual services were also identified, and the corresponding category and individual allowance standards were also identified.

### WISN results - staffing requirement

The results emanating from the study are based on documented annual workload from the ten (10) primary healthcare facilities in Kaduna North Local Government Area. The WISN results are presented in Table 2.

**Table 2.** WISN results for Kaduna North LGA.

Name of health facility	N/M available	N/M calculated	N/M gap/excess	N/M WISN ratio	CHW available	CHW calculated	CHW gap/excess	CHW WISN ratio
Doka (Zakari Isah) Primary Health Care Centre	1	3	-2	0.33	9	5	+4	1.80
Junction Road Primary Health Care Centre	1	3	-2	0.33	5	5	0	1.00
PHC Clinic Jos Road	1	1	0	1.00	1	2	-1	0.50
Primary Health Care Badarawa	1	3	-2	0.33	14	5	+9	2.80
Primary Health Care Centre Hayin Banki	1	11	-10	0.09	3	26	-23	0.12
Primary Health Care Centre Kabala	1	5	-4	0.20	5	13	-8	0.38
Primary Health Care Centre Unguwar Rimi	1	8	-7	0.13	5	20	-15	0.25
Primary Health Centre Unguwar Shanu	0	11	-11	0.00	4	24	-20	0.17
Primary Health Centre Mohammed Bello Tukur Memorial	1	3	-2	0.33	3	6	-3	0.50
Primary Health Centre Unguwar Sarki	1	6	-5	0.17	2	15	-13	0.13
T **otal for Kaduna North LGA**	**9**	**54**	**-45**		**51**	**121**	**-70**	

N/M – Nurse/Midwife; CHW – Community Health Worker Practitioners comprising of CHO – Community Health Officers; CHEW – Community Health Extension Workers; JCHEW – Junior Community Health Extension Workers; LGA – Local Government Area.

Kaduna North Local Government Area has only 17% of the Nurse/Midwife workforce it requires to provide primary health care services. Overall, the LGA requires about 54 Nurse/Midwives but currently has only 9 leaving a deficit of 45. All but one of the 10 PHCs have WISN ratios less than one (WISN < 1), indicating that the current number of Nurse/Midwife staff available is insufficient to cope with the workload. Primary Health Care Centre Hayin Banki has the lowest WISN ratio of 0.1, while there is no Nurse/Midwife available in Primary Health Centre Unguwar Shanu.

WISN results for CHWs; CHO, CHEW and JCHEW, also indicate a staffing shortage. Results provide an estimated requirement of 121, and an availability of 51 leaving a deficit of 70. CHWs are available in all assessed primary health care centres, with staffing surplus in two PHCs whose WISN ratios are above one (WISN >1); Doka (Zakari Isah) Primary Health Care Centre and Primary Health Care Badarawa. One PHC has the required number of CHWs, while the other seven PHCs have CHW staffing strength that is insufficient to cope with the work pressures.

## Discussion

Kaduna state has employed several workforce planning strategies that include traditional workforce estimation practices: health service target or disease-focused staffing estimation, health workforce to population ratio and population to facility staff ratio. Although these workforce planning strategies are useful, they are costly to implement and do not incorporate the complexities of the health system that affects health service seeking behaviours and service delivery. ^14^^,^^16^^–^^21^

This study applied the WISN methodology, and the literature suggests that this staffing estimating approach is most suitable for guiding the deployment of skilled frontline workers from places with fewer work pressures, to locations with higher work pressures, particularly in a resource-constrained environment. ^14^^,^^22^^–^^32^ Findings from this study revealed a gap in the number of Nurse/Midwives and CHWs available to provide primary health care services. Our study highlight variability in the availability of skilled HRH in these focal primary health care facilities. Nurses/Midwives are unavailable in all but one primary health facility with WISN ratios less than one, indicating that the current number of staff available is insufficient to cope with the workload. Conversely, CHWs are available in all assessed facilities, with a cumulative 13 staffing excesses in two PHCs; Doka (Zakari Isah) Primary Health Care Centre and Primary Health Care Badarawa, having WISN ratios greater than one; one PHC has the required number of CHWs, while the other seven PHCs have shortages of CHWs. Our findings are consistent with workload studies in Cross River and Rivers states, Nigeria as well as in Burkina Faso, ^14^^,^^15^^,^^33^ where there were shortages in the Nurse/Midwife cadre of the health workforce.

Our study presents several opportunities for the Kaduna state government. To the best of our knowledge, this study is the first attempt in the application of WISN to estimate staffing requirements in the state. As such, this study provides lessons on how to apply the WISN methodology. More importantly, it outlines the steps taken in establishing the WISN governance structure to drive ownership, knowledge transfer, follow-through on staffing decisions and prevents loss of institutional memory. For example, the study’s steering committee and technical task force were sub-sets of the broader state HRH TWG, which is responsible for guiding the state on sustainable development of HRH within the context of Government development priorities, increasing the availability and equitable distribution of skilled HRH amongst others. This HRH TWG is chaired by the Permanent Secretary and led by State Honourable Commissioner for Health. This study provides an evidence-base to redistribute staff from underutilized health facilities to locations that are experiencing high work pressures. The 13 surpluses CHWs should be redistributed to understaffed health facilities to increase coverage of primary health care services. A WISN scale-up study across the state primary health care structure is recommended to effectively utilize scare HRH towards increasing coverage and improving PHC services.

The WISN methodology estimates the number of health workers needed to cope with work pressure in the facility. However, there are a few assumptions that the health worker is available and not absent from duties, well-behaved, and that the administered health service is relying on established standards, amongst others. Regardless of having the right numbers in a facility, these workforce productivity and performance inhibiting factors could affect service delivery. A recommendation is for Kaduna state to routinely assess the PHC workforce productivity level to guide incorporating HRH management strategies with planning.

Our study had a few limitations and steps were taken to address them. There are no national or regional activity standards available across the different categories of services; clinical and non-clinical, for the primary health care level. For the study, the Expert Group was tasked with establishing the activity standards, and there were slight variations in the timings provided for the prioritized cadres. To address the marginals variation across the activity standards, an average was taken. Another limitation was with availability and quality of health care data at the facility level. At this level, challenges arose with the storage of paper registers. To address this challenge, we chose to compare and triangulate data with other sources; the national DHIS2. In cases where paper files/registers were unavailable during field trips to the facility due to poor storage facilities, we opted for data with the national DHIS2 data source.

## Conclusion

As countries strive towards achieving UHC, the need for equitable distribution of frontline health workers has become paramount. Historically, Kaduna state has relied on other ways of determining staffing requirements for health facilities; however, these have been unrealistic, costly, and difficult to implement. Our study applied the WISN methodology to estimate staffing requirements in Kaduna state government prioritized PHC facilities. The study highlights an acute shortage in Nurse/Midwives and CHW practitioners in these prioritized health facilities and provides evidence-based for determining staffing needs.

## Data availability

### Underlying data

Due to security restrictions, data cannot be made publicly available. Underlying data for this article are available on government approved health information systems. Annual health service statistics are available on the National Health Management Information System (NHMIS) - http://dhis2nigeria.org.ng, with access only available for users who have login details. Also, health workforce data is also available in the State Human Resources for Health Information System (HRH-IS).

For readers without access to these platforms, data may be requested by contacting the corresponding author via email ( bonkhi@gmail.com) or phone (+2348034118557) and access will be granted to in CSV format.

## Authors’ contribution

AIO, OT and HB conceptualized and designed the study, as well as coordinated its implementation. AIO coordinated data collection. AIO and OT analysed the data. AIO and OT drafted the initial manuscript. All authors read, reviewed, and approved the final version of the manuscript.
