# Determining staffing needs for improving primary health care service delivery in Kaduna State, Nigeria

**DOI:** 10.12688/f1000research.110039.2

**Published:** 2022-11-17

**Authors:** Agbonkhese I. Oaiya, Oluwabambi Tinuoye, Layi Olatawura, Hadiza Balarabe, Hamza Abubakar

**Affiliations:** 1 PATH, Abuja, Nigeria; 2 Abts Associates, FCT Abuja, Nigeria; 3 Health Strategy and Delivery Foundation, FCT Abuja, Nigeria; 4 Kaduna State Government House, Kaduna, Nigeria; 5 Kaduna State Primary Health Care Board, Kaduna, Nigeria

**Keywords:** Universal Health Coverage; Health Workforce; Human Resources for Health; Workload Indicators for Staffing Need; Nigeria; Service Delivery; Nurse; Midwife; Community Health Officer; Community Health Extension Worker; Junior Community Health Extension Worker.

## Abstract

**Background:** The equitable distribution of a skilled health workforce is critical to health service delivery. Kaduna state has taken significant steps to revamp the primary health care system to ensure access to health care for its populace. However, these investments are yet to yield the desired outcomes due to health workforce shortages and the inequitable distribution of those available.

**Methods:** A Workload Indicator for Staffing Need (WISN) study was conducted at Kaduna state's primary health care level. The study focused on estimating staffing requirements; Nurses/Midwives and Community Health Worker practitioners, Community Health Officers, Community Health Extension Workers, and Junior Community Health Extension Workers in all government-prioritised primary health care facilities. A total of ten focal primary health care facilities in Kaduna North Local Government Area (LGA) were included in the study.

**Results:** Findings from the study revealed a shortage of Nurses/Midwives and Community Health Workers across the study facilities. For the Nurse/Midwife staffing category, nine of the ten PHCs have a WISN ratio < 1, indicating that the number of staff in the Nurse/Midwife category is insufficient to cope with the workload. In two of the ten primary health care facilities, there is an excess in the number of CHWs available; a WISN ratio > 1 was calculated.

**Conclusion:** The WISN study highlights staffing needs in Kaduna State's government-prioritised primary health care facilities. This evidence establishes the basis for applying an evidence-based approach to determining staffing needs across the primary health care sector in the State to guide workforce planning strategies and future investments in the health sector. The World Health Organisation (WHO) WISN tool is useful for estimating staffing needs required to cope with workload pressures, particularly in a resource-constrained environment like Kaduna State.

## Introduction

The equitable distribution of resources, specifically Human Resources for Health (HRH), to meet the populace’s needs is critical for achieving Universal Health Coverage (UHC). However, this essential health system component is plagued with challenges in availability, distribution, skills, and retention. These challenges are prominent in developing countries like Nigeria, where shortages and inequitable health workforce distribution, poor HRH planning, inadequate recruitment exacerbated by a ban on workforce recruitment, and weak retention strategies are often encountered. ^1^^–^^4^ These frequently result in disparities in health worker densities by locality, limit access to health care services and inadvertently undermine the quality of care provided, all of which are associated with poor health outcomes.

In the last decade, Nigeria has developed some health system reforms aimed at improving national health indices. One such reform is the Primary Health Care (PHC) Minimum Service Package (MSP). ^5^^,^^6^ The MSP documents services to be provided by primary health facilities, articulates staffing norms and composition recommendations, and states what services each health worker cadre should provide. ^5^^,^^6^ The MSP was fundamental because it was birthed in the wake of the UHC push in Nigeria, and it targets primary health care, which is the entry point into the health system, where promotive, preventive, and curative services for uncomplicated minor ailments are provided in Nigeria. ^5^^–^^7^ The MSP stipulates that at the primary care level, the health workforce should include medical officers, nurses, midwives, community health practitioners, laboratory technicians, pharmacy technicians, health records assistants and environmental health officers. ^5^^–^^7^ However, these staffing standards have remained unmet. This may be due to the health workforce crisis that Nigeria is facing as well as the low government spending on health at national and sub-national levels. ^8^ In response to sub-optimal staffing patterns at the primary health care level, the Government of Nigeria developed a Task Shifting and Task Sharing (TSTS) policy that allows lower-skilled clinical staff to perform high-skilled clinical tasks following training. ^9^ However, this policy is fraught with implementation and monitoring challenges.

Kaduna is a State in Northern Nigeria, with a projected population of 8.98 million. ^10^ Kaduna is one of the 36+1 States in Nigeria that has made considerable investments in healthcare. ^11^ To attain UHC through community participation, as contained in the National Health Act, the State adopted and implemented the Ward Health System (WHS) for healthcare. ^5^^–^^7^^,^^12^ The WHS utilises the electoral wards as the elementary operational unit for PHC service delivery. This formed the basis for the State government’s investment in one PHC facility per ward. There are 255 wards in Kaduna state, and the State government prioritised one PHC facility in each ward. Since 2015, 255 PHC facilities have benefitted from the government’s investments in establishing and sustaining a multi-level administrative governance structure, improved infrastructure and provision of basic equipment and essential medicines, amongst others. However, the State has been unable to meet the staffing needs for these facilities as stipulated by the MSP because of its limited fiscal space for health spending. As such, the availability of a sufficient, skilled, and equitably distributed health workforce to serve the population in Kaduna has remained a challenge.

Considering Kaduna State’s HRH gaps and its inability to attain the MSP staffing norms, there is a need to employ an evidence-based staffing approach that could support the determination of adequate staffing norms in line with the existing challenges. One such method is the WISN method developed by WHO. This study aims to estimate the staffing requirements for healthcare delivery at focal primary health facilities by the health worker cadre in Kaduna North Local Government Area in Kaduna State, Nigeria, employing the WISN methodology.

## Methods

### Ethics approval and consent to participate

Written informed consent was obtained before data collection during the field visit through the Health Research Ethics Committee (HREC) of the Kaduna State Ministry of Health and had an approved registration number NHREC/17/03/2018.

The study employed the WISN methodology to determine staffing needs. WISN is designed by the WHO and supports the evidential determination of the number of health workers by cadre required to cope with the workload in a particular health facility. The WISN methodology considers several relevant components by health worker cadre that includes: (i) services delivered, (ii) the time it takes to deliver both clinical and non-clinical services, (iii) the total annual work time available to each Health Care Workers (HCW) cadre as well as (iv) retrospective annual service delivery statistics in the health facility. ^13^ Computation of the statistics from these components produces a determined number of HCWs by cadre required in each health facility.

### Scope of the study

The WISN study was completed in Kaduna North Local Government Area and included ten (10) primary health facilities. The study population were clinical health workers available and tasked with providing healthcare services to patients at these primary health facilities. These prioritised cadres are Nurses/Midwives and Community Health Workers (CHWs), comprising Community Health Officers (CHOs), Community Health Extension Workers (CHEWs), and Junior Community Health Extension Workers (JCHEWS). Health services such as Reproductive Maternal and Newborn Child Health (RMNCH), predominantly provided at the primary care level and make up most of the health facility visits in the LGA, were prioritised for the study.

### Establishing state governance structures

Three Technical Working Groups (TWGs): Steering Committee, Technical Task Force, and an Expert Group were inaugurated to conduct the study. These study groups were a subset of the State’s larger HRH TWG. Their objectives include providing HRH-related advisory and technical support to the State government to enable workforce development. The three group members were drawn from relevant Ministries, Departments and Agencies (MDA), health training institutions, Civil Society Organisations (CSO), health facilities and development partners. These groups were engaged to build local capacity and create utility for study results.

### Health facility inclusion criteria

Kaduna North Local Government Areas (LGA) was selected for convenience for this study. Consequently, all government-prioritised PHC facilities that had been in operation for at least one year before the time of the study were included. Kaduna North LGA is an urban area and one of the most densely populated areas in the State. The decision to include only government-prioritised PHC facilities is hinged on the significant investments made by the State government and donors in these facilities and a resultant increase in service utilisation rates.

### Data collection

After a review of relevant documents that include the Nigeria Task Shifting and Task Sharing (TSTS) policy, the MSP, Ward Minimum Healthcare Package (WMHCP) and the public service handbook, data collection tools were developed. Data on health service statistics, facility HRH composition, staff Available Work Time (AWT) and time spent by healthcare workers on clinical and non-clinical activities were collected and compared from both primary and secondary sources.

Primary data sources included health facility service delivery registers, staff registers, and expert judgments through an Expert Group discussion. Primary data collection lasted three weeks between June and July 2021. Secondary data sources included the Nigeria District Health Information System (DHIS2) and Kaduna State Primary Healthcare Board (KSPHCB) Human Resources for Health Information System (HRH-IS). The DHIS2 is the electronic instance of the National Health Management Information System (NHMIS), a paper-based mechanism aggregating all healthcare services delivered in a health facility.

For health service statistics, data for family planning, antenatal care, postnatal care, immunization, diarrhoea, pneumonia and malaria in children and adults from January to December 2019 were obtained from health facility registers and compared with those retrieved from the DHIS2. Further, during field visits, facility workforce data focusing on clinical cadres were obtained from health facility staff registers.

A multi-step approach was taken to obtain information on staff AWT (the total amount of time available to a HCW by cadre to perform daily tasks in a year, considering authorised and unauthorised absences).

Firstly, a desk review of relevant public service statutory policy, rules, and guidelines was conducted to obtain the total number of HCW’s work hours per day, work days per week, and authorised and unauthorised absences allowed within the State’s service. Finally, the Staff AWT was subsequently reviewed and approved by the study’s governance structure.

An Expert Group comprising 17 clinical experts were convened to obtain time spent on clinical and non-clinical activities by HCWs in the study’s cadres of interest. These experts were purposefully selected and included individuals who are members of the study’s cadre of interest, currently employed in the public service, and have at least 15 years of experience providing health care services at the primary care level. All experts in the group responded on time that it takes the prioritised health worker cadre to perform the selected activities to acceptable standards, and the mean value of their responses was utilised.

### Data analysis and interpretation

The data collected were analysed using MS Excel, consistent with the WISN methodology. Activity standards, clinical and non-clinical workload components, annual service delivery statistics and AWT for the prioritised cadre for each facility were included. To complete the computation, the data collected was defined and analysed as follows:

• Available Working Time: The time a health worker is available in one year to do their work, considering authorised and unauthorised absences. AWT in Days is the difference between Possible Working days in a year (PWD) and Non-working days in a year (authorised and unauthorised absences). ^13^^–^^15^

$$\mathrm{AWT}=A–\left(B+C+D+E\right)$$ (1.0)

Where in the formula:

AWT is the total staff available working time

A is the number of possible working days in a year

B is the number of days off for public holidays in a year

C is the number of days off for official leave in a year

D is the number of days off due to sick leave in a year

E is the number of days off due to casual leave, study or training leave and maternity leave in a year.

• Activity Standard: The time it takes an HCW of a particular cadre to deliver clinical and non-clinical services; core, individual and support activities. ^13^
• Standard Workload: The amount of work one HCW can perform in a year within a health service category. ^13^ It is calculated in unit time or rate of work by dividing AWT by the time taken to conduct the work or multiplying the AWT by the rate of working, respectively.
• Core health activities: The WISN methodology describes these as health service activities. They refer to activities performed by all cadre staff, and regular statistics are collected. ^13^ They are clinical and identified as clinical health services in this study.
• Support activities: According to the WISN methodology, these activities are performed by all members of the cadre, but routine statistics are not collected on them. ^13^ Although these activities contribute to service delivery, they are not clinically related and are identified as non-clinical in this study.
• Additional activities: These activities do not involve all staff of the same cadre, and routine statistics are also not collected on them. ^13^ For this study, they are categorised as non-clinical.
• Staff requirement for core health activities: This was calculated by taking the aggregate ratio of all annual core health services and standard workload for the identified clinical health services:
$$\sum_{i}^{n}\left(\frac{\mathrm{AWi}}{\mathrm{SWi}}\right)$$ (2.0)
  Core health activities i = 1,2,3 … n
  AWi = Annual statistics for each core clinical health service
  SWi = Standard Workload for each core clinical health service
• Staff requirement for support activities: A Category Allowance Standard (CAS), which is the percentage of the working time required to cope with all support activities, was estimated, and Category Allowance Factor (CAF) was calculated using:
$$\mathrm{CAF}=100/\left(100-\text{Total}\;\mathrm{CAS}\right)$$ (3.0)
  Staff requirement for individual activities: Individual Allowance Standard (IAS), which is the total number of hours per year needed to perform all additional activities undertaken by some HCWs, was also calculated. An Individual Allowance Factor (IAF) identifying the staffing requirement to undertake these workloads was estimated using the:
IAF=TotalIAS÷AWT (4.0)
• Total staffing requirement was calculated using the following:
$$\text{Total WISN Staff Requirement}=\mathrm{CAF}*\sum_{i}^{n}\left(\frac{\mathrm{AWi}}{\mathrm{SWi}}\right)+\mathrm{IAF}$$ (5.0)

WISN staffing results with fractions were handled as recommended by the WISN guide. ^13^ WISN differences and ratios were also calculated for each health facility. The WISN difference, which is calculated as the variance between the current staffing norm available by cadre and the computed staffing requirements and identifies staffing gaps or excesses by cadre. The WISN ratio represents the work pressure experienced by the HCW. A WISN ratio of > 1 indicates the availability of more HCWs than required to meet the facility workload.

## Results

### Clinical and non-clinical workload components and standards

Two categories of healthcare services were included in the study. The clinical health service forms the core health activities, while the non-clinical services comprise both support and additional services. The clinical core health services refer to activities directly related to service delivery performed by all prioritized cadres. ^13^^–^^15^ Support activities are part of the non-clinical category, and activities performed by the prioritized cadre are not directly related to patient care and usually involve all staff of the same cadre. ^13^^–^^15^ Additional activities are activities performed by these prioritized cadres that are not directly related to patient care and are undertaken by just a staff. ^13^^–^^15^

The workload and activity standards developed and validated by the expert group are presented in Table 1.

**Table 1.** Clinical and non-clinical workload components and service standards for primary level of care.

Service activity standards	Nurse/midwife	CHW	Unit
**A) Clinical health service – core health service activity standards**			
Antenatal care	10	10	Minutes/Client
Delivery	60	60	Minutes/Client
Postnatal care	14	19	Minutes/Client
Family planning (counselling)	18	17	Minutes/Client
Family planning (insertion of IUCD)	20	17	Minutes/Client
Family planning (insertion of an implant)	17	13	Minutes/Client
Family planning (injection)	7	8	Minutes/Client
Family planning (oral pills)	7	9	Minutes/Client
Immunization	9	11	Minutes/Client
Diarrhoea in U5 years old	14	13	Minutes/Client
Pneumonia in U5 years old	15	14	Minutes/Client
Confirmed uncomplicated malaria	18	21	Minutes/Client
**B) Non-clinical - support services activity standards**			
Community mobilisation and education	0	2	Hours/Week
Ward development committee meetings	51	50	Minutes/Month
Outreaches/community-based services	0	2	Hours/Week
Hand over/report writing	36	25	Minutes/Week
Staff meetings	38	33	Minutes/Week
Documentation on patients	30	30	Minutes/Day
Group health education	40	31	Minutes/Week
**C) Non-clinical - individual service activity standards**			
Supervision of students	60	60	Minutes/Day
General administration	50	66	Minutes/Day
Monthly report writing	50	69	Minutes/Month
Review meetings	2	3	Hours/Month
Mentoring of subordinates	44	36	Minutes/Day
Facility management meeting	51	39	Minutes/Month
Sterilisation of equipment	5	4	Minutes/Day

CHW – Community Health Worker Practitioners comprising of CHO – Community Health Officers; CHEW – Community Health Extension Workers; JCHEW – Junior Community Health Extension Workers.

A total of 26 health and non-health related services were identified in the state Primary Health Care level; of which 12 are clinical/core health services conducted by both Nurse/Midwife and CHW Practitioners. 14 non-clinical services; 7 support services and 7 individual services were also identified, and the corresponding category and individual allowance standards were also identified.

### WISN results - staffing requirement

The results emanating from the study are based on documented annual workload from the ten (10) primary healthcare facilities in Kaduna North Local Government Area. The WISN results are presented in Table 2.

**Table 2.** WISN results for Kaduna North LGA.

Name of health facility	N/M available	N/M calculated	N/M gap/excess	N/M WISN ratio	CHW available	CHW calculated	CHW gap/excess	CHW WISN ratio
Doka (Zakari Isah) Primary Health Care Centre	1	3	-2	0.33	9	5	+4	1.80
Junction Road Primary Health Care Centre	1	3	-2	0.33	5	5	0	1.00
PHC Clinic Jos Road	1	1	0	1.00	1	2	-1	0.50
Primary Health Care Badarawa	1	3	-2	0.33	14	5	+9	2.80
Primary Health Care Centre Hayin Banki	1	11	-10	0.09	3	26	-23	0.12
Primary Health Care Centre Kabala	1	5	-4	0.20	5	13	-8	0.38
Primary Health Care Centre Unguwar Rimi	1	8	-7	0.13	5	20	-15	0.25
Primary Health Centre Unguwar Shanu	0	11	-11	0.00	4	24	-20	0.17
Primary Health Centre Mohammed Bello Tukur Memorial	1	3	-2	0.33	3	6	-3	0.50
Primary Health Centre Unguwar Sarki	1	6	-5	0.17	2	15	-13	0.13
T **otal for Kaduna North LGA**	**9**	**54**	**-45**		**51**	**121**	**-70**	

N/M – Nurse/Midwife; CHW – Community Health Worker Practitioners comprising of CHO – Community Health Officers; CHEW – Community Health Extension Workers; JCHEW – Junior Community Health Extension Workers; LGA – Local Government Area.

Kaduna North Local Government Area has only 17% of the Nurse/Midwife workforce it requires to provide primary health care services. Overall, the LGA requires about 54 Nurses/Midwives but currently has only 9 leaving a deficit of 45. All but one of the 10 PHCs have WISN ratios of less than one (WISN < 1), indicating that the current number of Nurse/Midwife staff available is insufficient to cope with the workload. While there is no Nurse/Midwife available in Primary Health Centre Unguwar Shanu, Primary Health Care Centre Hayin Banki has the lowest WISN ratio of 0.09.

WISN results for CHWs: CHO, CHEW and JCHEW also indicate a staffing shortage. Results provide an estimated requirement of 121 and an availability of 51 leaving a deficit of 70. CHWs are available in all assessed primary health care centres, with staffing surplus in two PHCs whose WISN ratios are above one (WISN >1); Doka (Zakari Isah) Primary Health Care Centre and Primary Health Care Badarawa. One PHC has the required number of CHWs, while the other seven PHCs have CHW staffing strength that is insufficient to cope with the work pressures.

## Discussion

Kaduna state has employed several workforce planning strategies that include traditional workforce estimation practices: health service target or disease-focused staffing estimation, health workforce to population ratio and population to facility staff ratio. Although these workforce planning strategies are useful, they are costly to implement and do not incorporate the complexities of the health system that affects health service-seeking behaviours and service delivery. ^14^^,^^16^^–^^21^

This study applied the WISN methodology, and the literature suggests that this staffing estimating approach is most suitable for guiding the deployment of skilled frontline workers from places with fewer work pressures, to locations with higher work pressures, particularly in a resource-constrained environment. ^14^^,^^22^^–^^32^ Findings from this study revealed a gap in the number of Nurses/Midwives and CHWs available to provide primary health care services. Our study highlight variability in the availability of skilled HRH in these focal primary health care facilities. Nurses/Midwives are unavailable in all but one primary health facility with WISN ratios of less than one, indicating that the current number of staff available is insufficient to cope with the workload. Conversely, CHWs are available in all assessed facilities, with a cumulative 13 staffing excesses in two PHCs; Doka (Zakari Isah) Primary Health Care Centre and Primary Health Care Badarawa, having WISN ratios greater than one; one PHC has the required number of CHWs, while the other seven PHCs have shortages of CHWs. Our findings are consistent with workload studies in Cross River and Rivers states, Nigeria, as well as in Burkina Faso, ^14^^,^^15^^,^^33^^,^^34^ where there were shortages in the Nurse/Midwife cadre of the health workforce.

Our study presents several opportunities for the Kaduna state government. To the best of our knowledge, this study is the first attempt at applying WISN to estimate staffing requirements in the State. As such, this study provides lessons on how to apply the WISN methodology. More importantly, it outlines the steps taken in establishing the WISN governance structure to drive ownership, knowledge transfer, and follow-through on staffing decisions and prevents the loss of institutional memory. For example, the study’s steering committee and technical task force were sub-sets of the broader state HRH TWG. This study provides an evidence base to redistribute staff from underutilised health facilities to locations experiencing high work pressures. The 13 surpluses CHWs should be redistributed to understaffed health facilities to increase primary health care services coverage. For the Nurse/Midwife category, there is an acute shortage, with just nine available and a lack of 45. A WISN scale-up study across the state primary health care structure is recommended to effectively utilise scare HRH towards increasing coverage and improving PHC services.

The WISN methodology estimates the number of health workers needed to cope with work pressure in the facility. However, there are a few assumptions that the health worker is available and not absent from duties, well-behaved, and that the administered health service relies on established standards, amongst others. Regardless of having the right numbers in a facility, workforce productivity and performance-inhibiting factors could affect service delivery. A recommendation is for Kaduna state to routinely assess the PHC workforce productivity level to guide incorporating HRH management strategies with planning.

Our study had a few limitations, and steps were taken to address them. No national or regional activity standards are available across the different categories of services: clinical and non-clinical, for the primary health care level. For the study, the Expert Group was tasked with establishing the activity standards, and there were slight variations in the timings provided for the prioritised cadres. To address the marginal variation across the activity standards, an average was taken. Another limitation was with availability and quality of health service statistics at the facility level owing to paper storage challenges. To address this challenge, we compared the data with a secondary source; the DHIS2. In cases where paper registers were unavailable, we used data retrieved from the DHIS2.

## Conclusion

As countries strive toward achieving UHC, the need for equitable distribution of frontline health workers has become paramount. Historically, Kaduna State has relied on other ways of determining staffing requirements for health facilities; however, these have been unrealistic, costly, and difficult to implement. Our study applied the WISN methodology to estimate staffing requirements in Kaduna State government-prioritised PHC facilities. The study highlights an acute shortage of Nurses/Midwives and CHW practitioners in these prioritised health facilities and provides an evidence-based for determining staffing needs.

## Data availability

### Underlying data

Due to security restrictions, data cannot be made publicly available. The underlying data for this article are available on government-approved health information systems. Annual health service statistics are available on the National Health Management Information System (NHMIS) - http://dhis2nigeria.org.ng, with access only available for users with login details. Also, health workforce data is also available in the State Human Resources for Health Information System (HRH-IS).

For readers without access to these platforms, data may be requested by contacting the corresponding author via email ( bonkhi@gmail.com) or phone (+2348034118557), and access will be granted to the CSV format.

## Authors’ contribution

AIO, OT and HB conceptualised and designed the study, as well as coordinated its implementation. AIO coordinated data collection. AIO and OT analysed the data. AIO and OT drafted the initial manuscript. All authors read, reviewed, and approved the final version of the manuscript.
