# Supplementary material for: Determining staffing needs for improving primary health care service delivery in Kaduna State, Nigeria
Source: F1000Res. 2022 Nov 17;11:429. Originally published 2022 Apr 19. [Version 2] doi: 10.12688/f1000research.110039.2 (PMC9713056; doi:10.12688/f1000research.110039.2)
Supplement: Peer review history

# Peer review: Reviewer response for version 2

**DOI:** 10.5256/f1000research.140210.r156039

**Published:** 2023-01-20

**Authors:** Iboro Nelson

**Affiliations:** 1 Department of Economics, University of Uyo, Uyo, Nigeria; 2 USAID State2State Activity of DAI-Nigeria, Uyo, Nigeria; 3 Silverline Development Initiatives (SDI), Uyo, Nigeria

The authors have addressed the comments raised in the previously review sufficiently and satisfactorily.

Is the work clearly and accurately presented and does it cite the current literature?

Yes

If applicable, is the statistical analysis and its interpretation appropriate?

Yes

Are all the source data underlying the results available to ensure full reproducibility?

Yes

Is the study design appropriate and is the work technically sound?

Yes

Are the conclusions drawn adequately supported by the results?

Partly

Are sufficient details of methods and analysis provided to allow replication by others?

Yes

Reviewer Expertise:

Health Systems specifically health financing, human resource for health management

I confirm that I have read this submission and believe that I have an appropriate level of expertise to confirm that it is of an acceptable scientific standard.

# Peer review: Reviewer response for version 2

**DOI:** 10.5256/f1000research.140210.r156040

**Published:** 2022-11-30

**Authors:** Richard F Heller

**Affiliations:** 1 University of Newcastle, Newcastle, NSW, Australia

While the authors have not directly responded to all of the concerns raised in my original report, the revisions that have been made allow me to conclude that the paper is acceptable for indexing. I did ask for a discussion on the generalisability of the results to other settings, and while this has not been addressed directly, I think that others who wish to apply the results to their setting will find enough in the revised paper to allow them to do so.

Is the work clearly and accurately presented and does it cite the current literature?

Yes

If applicable, is the statistical analysis and its interpretation appropriate?

Yes

Are all the source data underlying the results available to ensure full reproducibility?

Yes

Is the study design appropriate and is the work technically sound?

Yes

Are the conclusions drawn adequately supported by the results?

Yes

Are sufficient details of methods and analysis provided to allow replication by others?

Partly

Reviewer Expertise:

Public health, health services, education

I confirm that I have read this submission and believe that I have an appropriate level of expertise to confirm that it is of an acceptable scientific standard.

# Peer review: Reviewer response for version 1

**DOI:** 10.5256/f1000research.121609.r135710

**Published:** 2022-05-20

**Authors:** Iboro Nelson

**Affiliations:** 1 Department of Economics, University of Uyo, Uyo, Nigeria; 2 USAID State2State Activity of DAI-Nigeria, Uyo, Nigeria; 3 Silverline Development Initiatives (SDI), Uyo, Nigeria

**Strength**: The work is a useful contribution to knowledge in the field of human resource for health management. Well-written in simple and understandable English devoid of jargons, the paper frames the problem statement in a lucid manner and situates it contextually. Key concepts such as WISN (Workload Indicators for Staffing Need) and the key steps, DHIS (District Health Information System), Task Shifting and Task Sharing are adequately defined and broken down to the readers’ understanding. The study limitations and mitigation strategies are also clearly discussed.

**Weakness**: However, in an attempt to present the evidence-base methodology of WISN approach for health workforce estimation as superior to the other approaches earlier adopted by the State such as disease-focused staffing estimation, and health workforce to population ratio and population to facility staff ratio, the study failed to make recommendations for a major of the study’s findings: the unavailability of Nurses/Midwives in all but one facility in the LGA (Local Government Areas). Besides, some grammatical error gaps which is usually characteristics of such work as this are highlighted for the authors’ consideration.

**General Conclusion**: The work has academic merit and is fit for indexing, howbeit a number of minor grammatical errors and changes to the article needs to be addressed and or amended.

Is the work clearly and accurately presented and does it cite the current literature?

Yes

If applicable, is the statistical analysis and its interpretation appropriate?

Yes

Are all the source data underlying the results available to ensure full reproducibility?

Yes

Is the study design appropriate and is the work technically sound?

Yes

Are the conclusions drawn adequately supported by the results?

Partly

Are sufficient details of methods and analysis provided to allow replication by others?

Yes

Reviewer Expertise:

Health Systems specifically health financing, human resource for health management

I confirm that I have read this submission and believe that I have an appropriate level of expertise to confirm that it is of an acceptable scientific standard, however I have significant reservations, as outlined above.

# Response

**Published:** 2022-10-23

**Authors:** Agbons Oaiya

Many thanks for your comments. The concerns raised have been addressed in the revised submission.

# Peer review: Reviewer response for version 1

**DOI:** 10.5256/f1000research.121609.r135712

**Published:** 2022-05-19

**Authors:** Pamela A. McQuide

**Affiliations:** 1 IntraHealth International, Chapel Hill, NC, USA

This is an important paper using the evidenced-based approach of the World Health Organization’s (WHO) Workload Indicator of Staffing Need to determine facility level staffing in primary health care (PHC) sites in Kaduna State in Nigeria. The authors have done well in applying this method and results for PHC. 

 There are a few considerations for the authors to improve the quality of the paper and a few small mistakes to correct.

 Small mistakes to correct:

1. Page 7 in the table on document on patients 3 minutes but the unit is missing. Is it per patient, per day etc. It would seem that 3 minutes per day to document on patients would not be sufficient.
2. Page 7, last paragraph states “Nurse/Midwives are unavailable in all but one primary health facility”. I think it should read NM are available in all but one primary health facility.
3. Page 6 second to last paragraph states Hayin Bank WISN ratio is .10 and in the Table 2, it says .09. It also says Hayin Bank has the lowest WISN ratio, but, Unguwar Sahnu WISN ratio is .0 because they have no Nurse/Midwives. This would be consistent in the abstract which states that 9/10 sties have WISN ratio <1.0 which would include the site with a WISN ratio of 0.0

Recommendations:

1. The paper did not indicate how the 10 PHC sites were selected. Were they part of the wards discussed in the introduction, randomly selected across Kaduna State or did they represent the different characteristics of sites in Kaduna State or another methodology?
2. It would be easier for the reader if the definitions for the types of teams (Steering Committee, Technical Team and Expert Group) are pulled together in the methods section and uses the definitions from WHO. It is fine that they are part of the larger HRH TWG group in this Kaduna State. The definitions in the paper are inconsistent with the WHO definitions in the WISN User Manual (WHO 2010) p. 17. Health service activities are performed by all members of a staff category and regular statistics collected; support activities are performed by all members of a staff category but regular statistics are not collected on them; and additional activities are performed by only a certain members of a staff category and regular statistics are not collected. Some of the support and additional activities can be clinical in nature but no statistics are available for them. 
3. Use of the term triangulated is not used as would be expected. It appears different data were used to fill in for missing data and not to validate the data used with different data sources (e.g. DHIS2 and primary data).
4. In the discussion it mentions that two other states in Nigeria had similar WISN results, i.e., Cross River and River State but it did not indicate if the health service activities and standards were comparable for PHC. You also indicated that there were no regional or national standards for PHC but it seems this could lead to adapting standards for the country.
5. It might be helpful to put in a small table showing the relationship with health service activities, support activities, and additional activities with the activity standard and the standards workload and allowance factor for the CAF and IAF. It is a little hard to follow in the paper and at times the terms used in the paper are inconsistent. For example, health service activities are sometimes called core activities. If you put all the definitions under methods section, then you can just discuss the results and not have to put in definitions in other parts of the paper. These definitions are important for setting up the study and not in the results section. Use the same terms through-out the paper or it gets confusing. 
6. Since the service statistics are not available, I cannot validate the results given in the paper. 
7. In the background section you mention previously staffing norms used for budgeting positions. It might be interesting to compare the staffing norms to the actual WISN results to see if they are comparable or if the WISN results could be used to develop new evidenced-based norms that can be used. 
8. In the Table 2, it gives staff available. Is the staff available consistent with budgeted positions for this facility? You might want to put in a comment in the paper about budgeted positions versus actual positions available. 

Thank you for using the evidenced based approach using WISN to estimate staffing needs for PHC.

Is the work clearly and accurately presented and does it cite the current literature?

Yes

If applicable, is the statistical analysis and its interpretation appropriate?

Yes

Are all the source data underlying the results available to ensure full reproducibility?

Partly

Is the study design appropriate and is the work technically sound?

Yes

Are the conclusions drawn adequately supported by the results?

Yes

Are sufficient details of methods and analysis provided to allow replication by others?

Partly

Reviewer Expertise:

NA

I confirm that I have read this submission and believe that I have an appropriate level of expertise to confirm that it is of an acceptable scientific standard, however I have significant reservations, as outlined above.

# Response

**Published:** 2022-10-23

**Authors:** Agbons Oaiya

Many thanks for your comments. The typos, grammatical errors and other concerns raised have been addressed in the revised submission.

# Peer review: Reviewer response for version 1

**DOI:** 10.5256/f1000research.121609.r135713

**Published:** 2022-04-26

**Authors:** Richard F Heller

**Affiliations:** 1 University of Newcastle, Newcastle, NSW, Australia

This paper is a thorough and well written report of a valuable project with important practical results. The findings indicate a gap between the required and provided staffing needs in primary health care in one part of one State in Nigeria. 

 I have three areas that I feel would improve the understanding of the paper.

 First, in the Methods, we learn that the data come from DHIS2 (District Health Information System), an electronic database, triangulated by field visits to health facilities. This is an appropriate method, but the term 'triangulation' does not give us adequate information about what was done. Were field visits made to each of the facilities, how was the data obtained, how was the 'triangulation' actually performed and how were discrepancies resolved?

 Second, the Methods give us five formulae, which each look fine to me, and relate to Table 2, which is really clear and presents the main results of the study. However, I can't see a description of how the formulae lead to the column headings (N/M and CHW available and calculated) in the Table. Clarifying this would make the paper more understandable.

 Finally, I would have liked to see a discussion of the generalisability of the findings. The choice of Kaduna North LGA (Local Government Areas) is described as purposive, and that is fine, but how representative might this particular LGA be to other settings. Government prioritized health facilities within the LGA were chosen for the study, but how representative might these be to to generality of health facilities.

 I think that it should be quite easy to resolve the issues I have highlighted, which are only matters of presentation rather than questions about the validity of the research.

Is the work clearly and accurately presented and does it cite the current literature?

Yes

If applicable, is the statistical analysis and its interpretation appropriate?

Yes

Are all the source data underlying the results available to ensure full reproducibility?

Yes

Is the study design appropriate and is the work technically sound?

Yes

Are the conclusions drawn adequately supported by the results?

Yes

Are sufficient details of methods and analysis provided to allow replication by others?

Partly

Reviewer Expertise:

Public health, health services, education

I confirm that I have read this submission and believe that I have an appropriate level of expertise to confirm that it is of an acceptable scientific standard, however I have significant reservations, as outlined above.

# Response

**Published:** 2022-10-23

**Authors:** Agbons Oaiya

Many thanks for your insightful comments. You concerns have been taken care of in the revised manuscript.
